# New Holistic Conceptual Framework for the Assessment of the Performance of Photocatalytic Pavement

**DOI:** 10.3389/fchem.2020.00743

**Published:** 2020-09-16

**Authors:** Eva Jimenez-Relinque, Francisco Rubiano, Ramon Hingorani, Maria Grande, Angel Castillo, Roman Nevshupa, Marta Castellote

**Affiliations:** Spanish National Research Council, Eduardo Torroja Institute of Construction Sciences (IETcc-CSIC), Madrid, Spain

**Keywords:** photocatalytic pavement, assessment, performance, NO_x_ abatement, indicator, conformity, LIFE-photoscaling

## Abstract

Despite serious health and environmental burdens associated with air pollution by NO_x_, the emission ceilings have been systematically exceeded in big European cities for several years. Photocatalytic technology can be an efficient solution for the removal of chemical air pollutants. Because diesel engine exhaust is the main source of NO_x_ emissions, the application of a photocatalyst onto road pavement appears to be an effective NO_x_ abatement method due to the large surface area, proximity to the emission source, and relatively good solar irradiance. Several laboratory-scale studies provided evidence demonstrating that most harmful contaminants can be readily mineralized. Furthermore, several projects were aiming to scale up this technology to pilot and real scales. Although the photocatalytic performances of selected materials in real urban environments were determined in some of these studies, the data are not conclusive for evaluating the overall performance because other material characteristics relevant to their functionality were not assessed. The lack of conformity criteria suitable for the evaluation of the overall performance of photocatalytic pavement under real operational conditions has generated skepticism and mistrust among public authorities and relevant stakeholders, which constrains the widespread implementation of this promising technology. In this context, the project LIFE–Photoscaling was focused on developing a new holistic conceptual framework to assess the photocatalytic pavement performance using the decision tool “*Photoscaling Decision Maker*” based on a set of quantitative indicators. For this purpose, a large volume of data obtained for 10 types of photocatalytic pavement materials was systemized on both the laboratory and pilot plant scales and three main indicators were defined: (1) photocatalytic performance effectiveness, (2) intrinsic performance, and (3) undesired secondary effects. Each top-level indicator includes several low-level subindicators associated with specific material characteristics. Finally, the ranges of the main indicators and subindicators and methods for their assessment were determined. These methods include standard, adopted, and original characterization techniques, which were selected based on the criteria such as simplicity, cost- and time-effectiveness, and relevance regarding the operational conditions.

## Introduction

Air pollution is a global threat with a large impact on human health and ecosystems (EEA, 2019). In 2018, the First Global Conference on Air Pollution and Health organized by the World Health Organization (WHO) urged to act against air pollution, both indoor and outdoor, which is responsible for ~7 million deaths per year worldwide. In Europe, air pollution is the largest environmental health risk (HEI, 2018), highlighting the urgent need to reduce the levels of air contamination.

In this context, photocatalytic construction materials can be an efficient solution for the removal of chemical air pollutants. The photocatalytic properties of a construction material are based on the addition of semiconductor nanoparticles, mainly TiO_2_, to their volume or surface. Photocatalytic nanoparticles are activated by absorbing solar light in the UVA region (Serpone and Pelizzetti, 1989; Fujishima et al., 1999; Herrmann, 1999). For the abatement of NO_X_ pollution, which mainly originates from diesel engine exhaust, the photocatalytic material should be placed as close to the emission source as possible. Hence, photocatalytic pavements appear to be effective NO_x_ abatement methods due to their proximity to the pollutant source, large surface area, and, depending on the location, relatively good solar irradiance (Jones and Watts, 1997; Chen et al., 2007; Ballari et al., 2010).

The results of several laboratory studies proved that photocatalysis effectively deactivates and mineralizes most of the harmful contaminants such as NO_x_ (Cassar, 2004; Chen and Poon, 2009a,b; Bengtsson and Castellote, 2010; Folli et al., 2010; Lucas et al., 2013; Bloh et al., 2014; Jimenez-Relinque et al., 2015; Mendoza et al., 2015; Mills and Elouali, 2015; Hernández Rodríguez et al., 2016; Mothes et al., 2016; Jimenez-Relinque and Castellote, 2019b; Chen et al., 2020). Furthermore, this technology has already been tested on the pilot and real scales in several projects; the results indicate photocatalytic effectiveness (Beeldens et al., 2007; Maggos et al., 2007, 2008; Chen and Chu, 2011; Boonen and Beeldens, 2013; Suarez et al., 2014; Folli et al., 2015; Gallus et al., 2015; George et al., 2016; Jiménez-Relinque et al., 2019; Cordero et al., 2020). However, the information obtained in these studies is not conclusive for evaluating the overall performances of photocatalytic pavements because other material characteristics relevant to their functionality, such as the durability, or potential undesirable secondary effects were not assessed.

In fact, precautions should be taken to prevent photocatalytic NO_x_ oxidation from generating dangerous or toxic intermediate products (Langridge et al., 2009; Monge et al., 2010; Bloh et al., 2014). Furthermore, the introduction of large amounts of nanofunctionalized materials to the urban environment can have several negative effects such as the release of nanoparticles into the air (Zhu et al., 2004; Pacheco-Torgal and Jalali, 2011; Jayapalan et al., 2013) or aquatic ecosystems (Kägi et al., 2008; Olabarrieta et al., 2012; Al-Kattan et al., 2013; Zuin et al., 2014; Shandilya et al., 2015; Bossa et al., 2017). It should be highlighted that despite the availability of various photocatalytic construction materials on the market, there are currently no clear guidelines or specific methods that can be used to evaluate their effects, with the exception of the nitrate selectivity during NO conversion (Bloh et al., 2014; Patzsch et al., 2017). The lack of a holistic conceptual framework and conformity criteria, which are required for the knowledge-based evaluation of the overall performances of photocatalytic pavements under real operation conditions, has generated skepticism and mistrust among public authorities and relevant stakeholders, which constrains the widespread implementation of this promising technology.

These problems were addressed in the project LIFE-Photoscaling (LIFE 13/ENV/ES/001221). The purpose of this project was to build the scientific groundwork for the holistic evaluation of the overall performance of photocatalytic pavement and the required procedures and to establish a set of conformity criteria. The new conceptual framework and its application to 10 photocatalytic materials belonging to three main groups, that is, coatings obtained from slurries and suspensions, bulk cementitious materials, and ceramic tiles, based on a user-friendly decision-making tool, are presented in this article. The framework of this dynamic decision-making tool enables the incorporation of new materials and new data for already selected materials as well as the continuous adjustment of both scores and importance factors (IFs) in accordance with the increases in the available data and knowledge regarding relevant processes.

The remainder of the paper is organized as follows: in the experimental section, the methodology, design of experiment, and flux of information between the different stages are explained. Afterward, the laboratory and outdoor tests in the platforms are detailed, minimum conformity parameters (subindicators) are established, and results of experimental tests are discussed and critically analyzed. After establishing the IFs and weights for main indicators and subindicators, the examples of application of this methodology for assessment of various products are described.

## Experimental

### Methodology and Experimental Design

Photocatalytic pavements must reconcile the functions of ordinary pavements, i.e., mechanical strength, durability under adverse environmental conditions, adequate friction and wear properties, and permeability, with advanced properties such as air depollution (effectiveness for NO_x_; volatile organic compounds, VOCs; and mineralization of other chemical pollutants) and self-cleaning. Furthermore, photocatalytic pavements should not be the source of other types of environmental pollution such as nanoparticle aerosols, water pollution by leached metals, and eutrophication related to nitrate formation by the oxidation of nitrogen oxides. In addition, aesthetical aspects of the pavement are important in urban environments; therefore, adding a photocatalytic coating or photocatalytic compound to asphalt or cement paste should not substantially change the pavement color.

**Figure 1:**
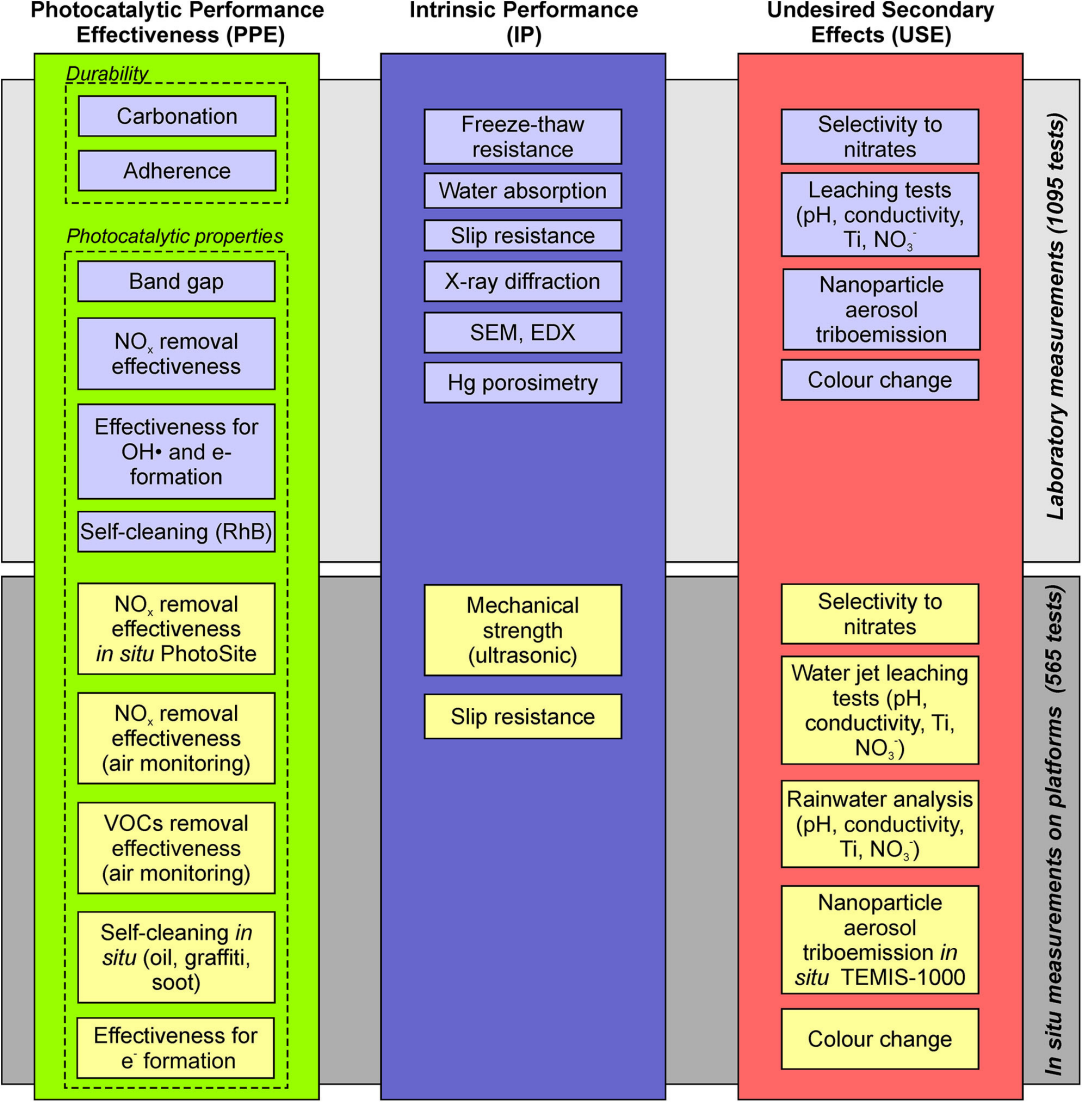
Holistic approach for the assessment of the functional performance of photocatalytic pavement based on three main indicators.

Therefore, the approach for a holistic assessment of the functional performance of the photocatalytic pavement leads to the establishment of three main indicators (Figure 1): photocatalytic performance effectiveness (PPE), intrinsic performance (IP), and undesired secondary effects (USE). By considering multiple aspects of each main performance indicator, a set of subindicators was defined, which can be used to characterize the photocatalytic effectiveness, photocatalytic durability, mechanical strength, tribological properties, self-cleaning, leaching, eutrophication, and potential for nanoparticle aerosol generation due to triboemission. Subsequently, a comprehensive experimental program was established to evaluate the abovementioned parameters on both the laboratory and pilot platform scales. Various photocatalytic materials and reference materials without photocatalytic properties were placed on the banks of two outdoor platforms and characterized or monitored *in situ* using traditional methods and techniques. Several cores were extracted from the photocatalytic materials on the platforms to conduct laboratory tests. The cores were extracted at different times to study the effect of material aging.

**Figure 2:**
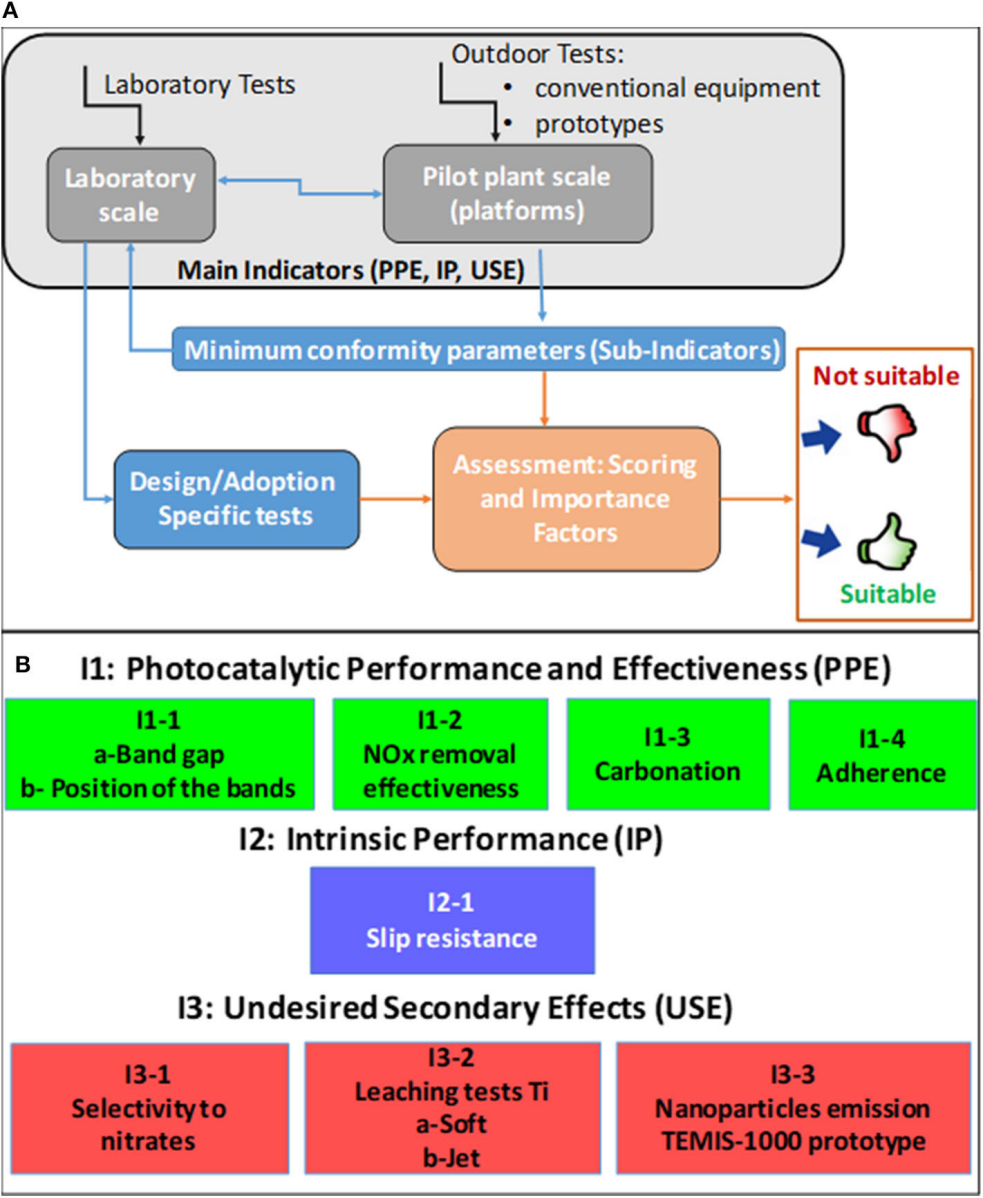
**(A)** Data flowchart used to refine the exhaustive set of conformity criteria for photocatalytic pavements. **(B)** Refined set of conformity criteria for photocatalytic pavements consisting of main and subindicators.

Although the complete set of parameters provides insights into the performance of the photocatalytic pavement from various perspectives, measuring all parameters is very costly and time-consuming and thus limits the applicability of this methodology in practice. Hence, a model refinement was performed to compromise between a sufficient volume of information on various aspects of the photocatalytic pavement performance and the model usefulness. For this purpose, the following criteria were defined: (i) using as few parameters as possible, (ii) choosing only the most important conformity criteria, and (iii) preferential selection of tests that can be conducted in the laboratory using the simplest techniques. These aspects will be explained in more detail in section Minimum conformity parameters. The schematic drawing of the procedure used to refine the initial exhaustive set of conformity criteria and establish the subindicators is shown in Figure 2A. The final refined set of subindicators is displayed in Figure 2B.

To quantify the material performance, the measurable parameters corresponding to each subindicator were scored from 1 (less favorable) to 4 (most favorable).

Additionally, a score of zero was used to define the parameter range which may be incompatible with an application. If a product receives a zero in any of the subindicators, it is not suitable for practical use.

For the global assessment of the material performance, different weights were assigned to the subindicators depending on their relevance. In the first step, the sum of the points obtained for the different subindicators was normalized by using the maximum score for the corresponding main indicator, yielding the score/indicator. Subsequently, the global assessment value was obtained by summing the scores/indicators, which were multiplied by the IF for each main indicator. The set comprising the partial scores for the subindicators and global assessment value represents the conformity criteria for a photocatalytic pavement material.

### Photocatalytic Materials

Ten photocatalytic materials (“material” refers to the combination of a commercial photocatalytic product and pavement substrate) were chosen as a representative set of prevailing commercially available photocatalytic pavements. The materials include open-graded asphalt pavements (24.6% of air voids according to UNE-EN 12697-08:2008) with photocatalytic coatings in the form of slurries or suspensions. In addition, precast cementitious tiles coated with photocatalytic suspensions or containing the photocatalyst in the bulk were used in this study and photocatalytic ceramic tiles were tested. For each of the 10 materials (numbered from 1 to 10), reference specimens were fabricated for comparison. Although the compositions and characteristics of the photocatalytic materials cannot be disclosed in this paper due to confidentiality reasons, many materials and substrates were included in this study, which enabled the establishment of a reliable conceptual framework for the performance assessment of various classes of photocatalytic pavements.

### Laboratory Tests

At the laboratory scale, characterization tests, and experiments were conducted on the cores extracted from the banks of the platforms at different times, using traditional and new techniques. In case of the PPE indicator (Figure 2B), the band gap was determined with diffuse reflectance spectroscopy; the NO_x_ removal effectiveness was measured according to the standard ISO 22197-1:2007; the self-cleaning effectiveness was evaluated according to the standard UNI 11259:2008 using Rhodamine B (RhB). Methods using photocatalytic indicator probes were employed to determine the amount of generated hydroxyl radicals and free electrons (Jimenez-Relinque and Castellote, 2015, 2018, 2019a,b).

To assess the adhesion strength of photocatalytic coatings, a modification of the cross-cut test described in the International Standard of Paints and Varnishes (ISO 2409:2013) was developed. A photocatalytic coating was applied to the parallelepiped specimens with dimensions of 10 × 5 cm. The specimens were prepared 16 h before the test and kept at 23°C and a relative humidity of 50%. The samples were manually scratched using a multiblade cutter with a V-shaped 2-mm-deep cutting edge. The scratches followed a lattice pattern with several horizontal and vertical cuts. The tool was held perpendicular to the surface, and incisions were made by applying a constant force. Spaces of 1–2 mm were left between the perpendicular incisions to create well-defined intersections. Subsequently, an adhesive tape was used to detach the loosened coating fragments. The mass of the particles adhering to the tape, *M*_*a*_ (g/m^2^), was used as a criterion of the adhesive strength.

To evaluate the susceptibility of various materials to carbonation, several laboratory tests were carried out and the results were compared with data obtained for outdoor platforms (section Pilot Plant Tests). Based on these tentative experiments, an accelerated carbonation test with a simple experimental setup was carried out in a 100% CO_2_ atmosphere. The samples were kept in a controlled atmosphere with a relative humidity of 65% to maintain a stable water content for 1 week. Subsequently, all sides of the parallelepiped samples, except for the side with the photocatalytic coating, were coated with a sealant to avoid the bulk carbonation of the substrate. The samples were weighted and placed in a sealed chamber filled with CO_2_ gas. To compensate for absorption, the inlet of the chamber was purged with CO_2_ gas, while a non-return valve to the atmosphere was connected to the outlet. During the test, a relative humidity of 65% was maintained, which favors carbonation. At certain points, the samples were extracted for weighing. Subsequently, they were returned to the chamber and the abovementioned procedure was repeated.

In case of the IP indicator (Figure 2B), the freeze–thaw resistance was evaluated based on a modified CEN/TR 15177: 2006 procedure. Water absorption was measured according to the method described in De Rincón et al. (2007). The slip resistance was measured using a pendulum tester according to the EN 14231 standard. The chemical and structural analyses of the samples were carried out using X-ray diffraction, mercury intrusion porosimetry, and electronic microscopy (SEM/BSE with EDX).

In the case of the USE indicator, the selectivity of the photocatalytic reaction to nitrates was determined with the NO_x_ removal effectiveness tests based on the formation of NO_2_ during the photocatalytic process.

The amount of Ti leaching from the samples was measured according to two different methods, and the pH, conductivity, and nitrate, and Ti contents of the resulting leachates were determined. The method that reproduced the rainwater behavior of the outdoor slabs the best was the CEN/TS 16637-2 standard: Construction products–Assessment of the release of dangerous substances. Part 2: Horizontal dynamic surface leaching test. This standard specifies a dynamic surface leaching test for the determination of the surface-dependent release of substances from monolithic, plate-like, or sheet-like construction products or granular construction products with low hydraulic conductivity under standardized conditions. The test portion of the product was placed in a reactor/leaching vessel, and the exposed surface was completely submerged in a leachant, in this case pH-neutral demineralized water, with a volume of liquid to surface area ratio of 80 ± 10 L/m^2^ at a temperature ranging from 19 to 25°C, which was renewed at predetermined time intervals. This test method produces eluates, which are subsequently analyzed. In all cases, Ti was detected during the first steps. Subsequently, a short version of the standard was adopted, reducing the experiment time from 56 to 9 days. The method that best reproduced the behaviors of the slabs during waterjet cleaning was the home-developed method called “irrigation method.” The sample to be tested was placed in a leaching vessel on the top of a supporting structure, with the surface being exposed 7–8 cm from the bottom of the vessel. Under a flow rate of 4.16 × 10^−6^ m^3^/s, pure water was sprayed on each sample for 1 min. The test conditions were as follows: pH-neutral demineralized water as leachant, temperature of 19–25°C, flow rate of 4.16 × 10^−6^ m^3^/s, and L/A ratio of 50 ± 10 L/m^2^.

**Figure 3:**
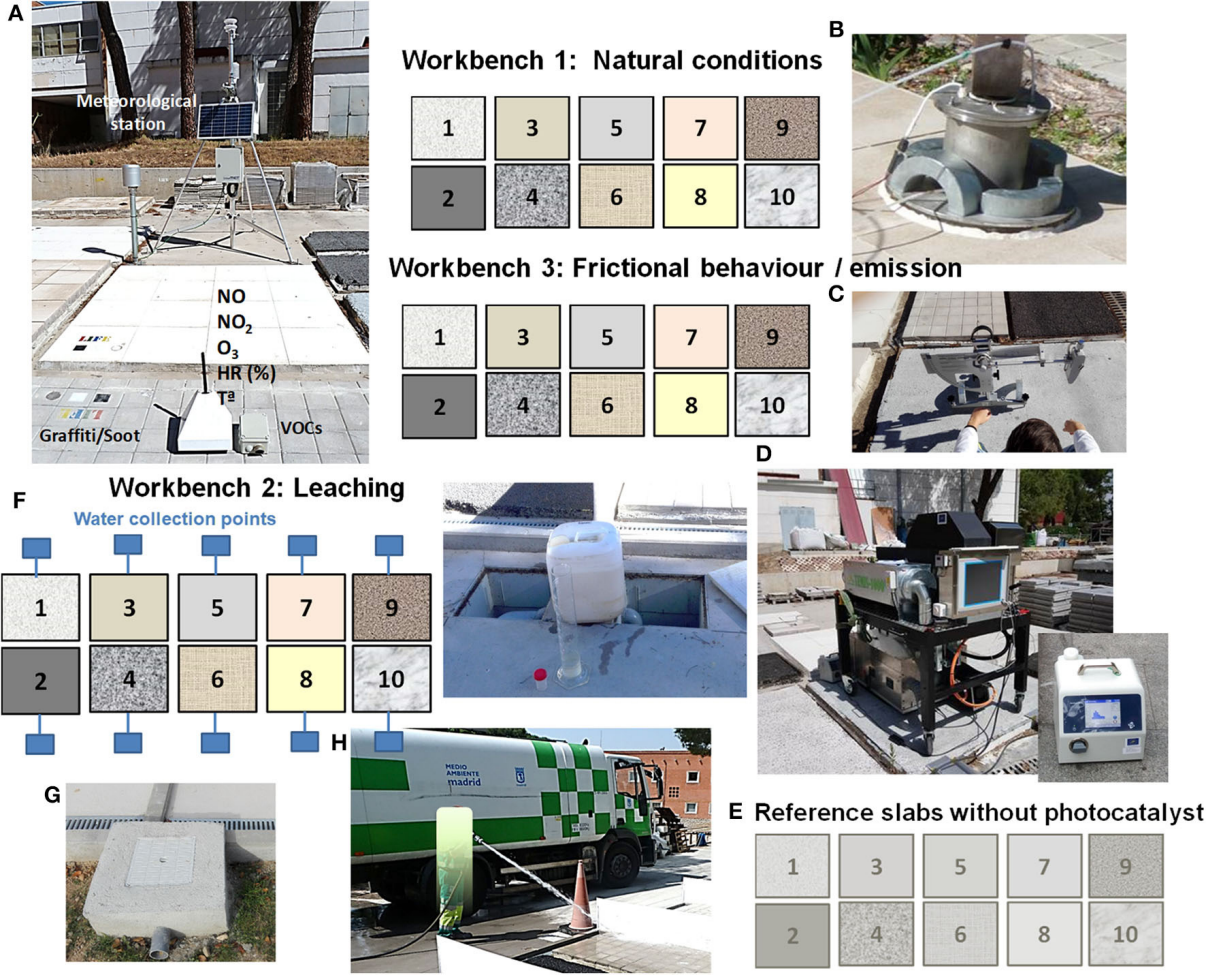
Schematic drawing of the platforms comprising three workbenches with active materials and a reference workbench. Photographs: **(A)** meteorological station for the continuous *in situ* monitoring of ambient parameters, view of slabs with graffiti and soot for degradation, and measurement of air pollutants using AQmesh low-cost sensors for NO_X_ and photoionizers for VOCs; **(B)** *in situ* measurement of the photocatalytic effectiveness using a PhotonSite device; **(C)** measurement of the slip resistance with a pendulum tester; **(D)** *in situ* measurement of the nanoparticle aerosols generated due to tire abrasion using the test rig TEMIS-1000; **(E)** SMSP nanoparticle sizer; **(F)** sink for the collection of rainwater; **(G)** a view of the water collector system; and **(H)** waterjet washing of the samples.

The nanoparticle emissions were analyzed using the experimental system TEMIS-1000, which was designed and constructed in the project, and the original procedure and analytical method to calculate the triboemission rate (Nevshupa et al., 2020a; Figure 3D). The test rig consisted of an aerosol-tight case, which was placed on top of the pavement or the sample of the pavement to be characterized. A soft rubber tape or similar material was used to seal the joint between the enclosure and pavement or surface of the sample holder. A working wheel with a tire, which performed rotation and reciprocating motion forth and back along the *y* axis, was placed inside the case. A well-controlled dead load was applied to the wheel mechanism to produce an adequate contact pressure between the wheel and pavement. Before the measurement, the enclosure was cleaned using wipes and organic solvents to remove deposited particles and organic contaminants from internal surfaces. When the aerosol concentration of the ambient air cannot be controlled, for example, during *in situ* measurements on a street, an equilibrium between the aerosol concentration inside and outside the case must be achieved before starting the test to avoid background noise, which can affect the results. For this purpose, the case was left open for at least 10 min prior to the start of the experiment. After closing the case, the residual aerosol concentrations were measured for 10 min or longer to obtain a benchmark. Subsequently, the setup was set in motion (rotation only or a combination of rotation and reciprocating motion), while the aerosol concentration was continuously measured using a nanoparticle sizer (SMSP, Figure 3E). After the completion of the required cycles, the test stopped automatically, but the measurement of the aerosol concentration continued for at least 40 min to obtain data on the transient concentration decay, which is necessary for the determination of particle deposition velocities. The experiment was repeated at least three times on different surface zones of the same sample. The characteristics of the prototype and experimental conditions of the tests for the conformity assessment are listed in Table 1. The test conditions for the asphalt samples were milder than those used for cement tiles to maintain a reasonable degree of wear.

**Table 1:** Characteristics of the prototype TEMIS-1000 and experimental conditions for conformity assessment for samples of tiles and open asphalt.

	**Tiles**	**Asphalt**
Tire type	3.5–8 Michelin S83	
Tire pressure (bar)	2.5	
Rotation velocity (rps)	0.4	
Reciprocating motion:		
Distance (mm)	50	
Half period (s)	200	
Number of cycles	10	6
Normal load (N)	200	400

The variations in the colors due to the application of photocatalytic products were assessed as a function of the change of the color of the CIELAB color space using the parameter ΔE (Equation 1). A portable spectrophotometer (CM-2300d, Konika Minolta) was used to obtain the color space parameters L^*^, a^*^, and b^*^.

(1) $$\begin{array}{l} \Delta E^{*}=\sqrt{{\left(L_{2}^{*}-L_{1}^{*}\right)}^{2}+\;{\left(a_{2}^{*}-a_{1}^{*}\right)}^{2}+{\left(a_{2}^{*}-a_{1}^{*}\right)}^{2}}, \end{array}$$

where subindexes 1 and 2 correspond to the values of the photocatalytic sample and reference (non-photocatalytic sample), respectively.

### Pilot Plant Tests

Two pilot-scale photocatalytic demonstration platforms were constructed (more information: www.lifephotoscaling.eu). Each platform consisted of three workbenches intended for the characterization of the leaching, abrasion, and aging properties of 10 slabs of photocatalytically active materials and corresponding reference materials, i.e., substrates without any photocatalytic coating. The platforms were placed in two different environments: one in the north of Madrid and the other in Arganda del Rey in the outskirts of Madrid. Both platforms were equipped with a meteorological station (Figure 3A). A schematic drawing of the platforms is shown in Figure 3.

The materials were characterized *in situ* on the platforms for 24 months (January 2016 to February 2018).

The main features of the pilot plant tests for the holistic assessment of the PPE main indicator can be summarized as follows: The NO_x_ effectiveness of the materials under natural outdoor conditions was evaluated using four AQmesh low-cost sensor pods (Figure 3A). After proper calibration, these sensors were placed on the ground level on different slabs for the continuous monitoring of the NO and NO_2_ concentrations. The calibration procedure and detailed results are described elsewhere (Cordero et al., 2020). To measure the abatement effectiveness of the VOCs under outdoor conditions, portable photoionizers were used (Figure 3A). They were placed onto the photocatalytic and reference slabs, and the pollutant removal effectiveness was determined based on the difference in the concentrations measured on the active and reference materials. The self-cleaning effectiveness was analyzed by monitoring the color evolution of the paints of different colors applied onto the materials to simulate graffiti, used engine oil, engine exhaust soot, and chewing gum (Figure 3A). In addition, similar to the laboratory tests, the RhB content and capacity of photocatalytic materials to generate free electrons and hydroxyl radicals were determined. The *in situ* NO_x_ removal effectiveness was assessed with a PhotonSite device; the procedure is explained in detail elsewhere (Jiménez-Relinque et al., 2019) (Figure 3B).

In the case of the IP indicator, the mechanical strength was assessed using ultrasound measurements defined in ASTM C597. The measurements were conducted with an indirect procedure after positioning the transducers directly on the slab substrates with the inner ends spaced at 10 cm. The slip resistance of the slabs was assessed using a standard pendulum test (EN 14231; Figure 3C).

Concerning the USE indicator, the nitrate selectivity was determined from *in situ* NO_x_ measurements using the original PhotonSite device. To analyze the leaching of potential contaminants, the rainwater is collected separately from each slab and the Ti and ${\text{NO}}_{3}^{-}$ contents, pH, and conductivity were analyzed (Figures 3F,G). In addition, the slabs were cleaned with a waterjet following the standard street cleaning procedure in Madrid (Figure 3H). The nanoparticle triboemission was studied using accelerated abrasion tests. A tire was employed and slid against the pavement slabs *in situ* using the original test rig TEMIS-1000 (Figure 3D). Finally, the color variations of the photocatalytic materials were measured by using Equation (1) (see section Laboratory Tests) and comparing the colors before and after the triboemission test.

In summary, in addition to the continuous monitoring of the environmental parameters, NO_x_ and VOC concentrations, 33 different types of tests were carried out, yielding 565 *in situ* tests and 1,095 laboratory analyses/trials. The volume of experimental data allowed the investigation of the most important aspects of the photocatalytic pavement performance. Given the enormous volume of experimental data, only the findings relevant to establishing a holistic conceptual approach for the assessment of the photocatalytic pavement performance and corresponding quantitative conformity criteria are summarized in this paper. Additional data can be found in the literature (Jiménez-Relinque et al., 2019, 2020; Cordero et al., 2020; Hingorani et al., 2020; Nevshupa et al., 2020b) or on the project website (www.lifephotoscaling.eu).

## Minimum Conformity Parameters

In this section, the different subindicators, their relevance, and the experimental methods to that were used to determine relevant parameters and their classification are presented. The score ranges assigned to these parameters are summarized in Table 2.

**Table 2:** Scores assigned to different ranges.

**Subindicator**	**Parameter**		**Score**				
			**0**	**1**	**2**	**3**	**4**
I1-1-a	*E_*c*_*/*E_*V*_* (V/V)			≥−0.33/<2.00	≥−0.33/≥2.00–<2.72	≥−0.33/≥2.72	<−0.33/≥2.72
I1-1-b	E_g_ (eV)			>3.26	2.75–3.26	2.5–2.75	<2.5
I1-2	*R_*NOx*_* (%)		<5	5–10	10–20	20–30	>30
I1-3	*M_*A*_* (g/m^2^)			>12	4–12	0–4	0
I1-4	*M_*C*_* (g/m^2^)			>1,500	500–1,500	0–500	0
I2-1	*Sr*		≤15	15–45	45–70	70–100	>100
I3-1-a	*M_*L*_* (mg/m^2^)	Soft	>10	5–10	1–5	0–1	0
I3-1-b		Jet	>15	7–15	1–7	0–1	0
I3-2	N^*^_te_ (1,000 #/cm^3^)	OGA^a^	>80	30–80	8–30	0–8	<0
		CT^b^	≥1.2	1.2–0.8	0.4–0.8	0–0.4	<0
I3-3	${\text{S}}_{{\text{NO}3}^{-}}$		<0.75	0.75–0.85	0.85–0.9	0.9–0.95	>0.95

* *N_te_ as function of the type of the pavement substrate (N_te_ is given in units 1,000/cm^3^)*.

a *OGA, open-graded asphalt*;

b *CT, cement tiles*.

### Main Indicator I1: Photocatalytic Performance Effectiveness

#### Subindicator I1-1: Band Gap (eV) and Position of the Bands (V)

**Figure 4:**
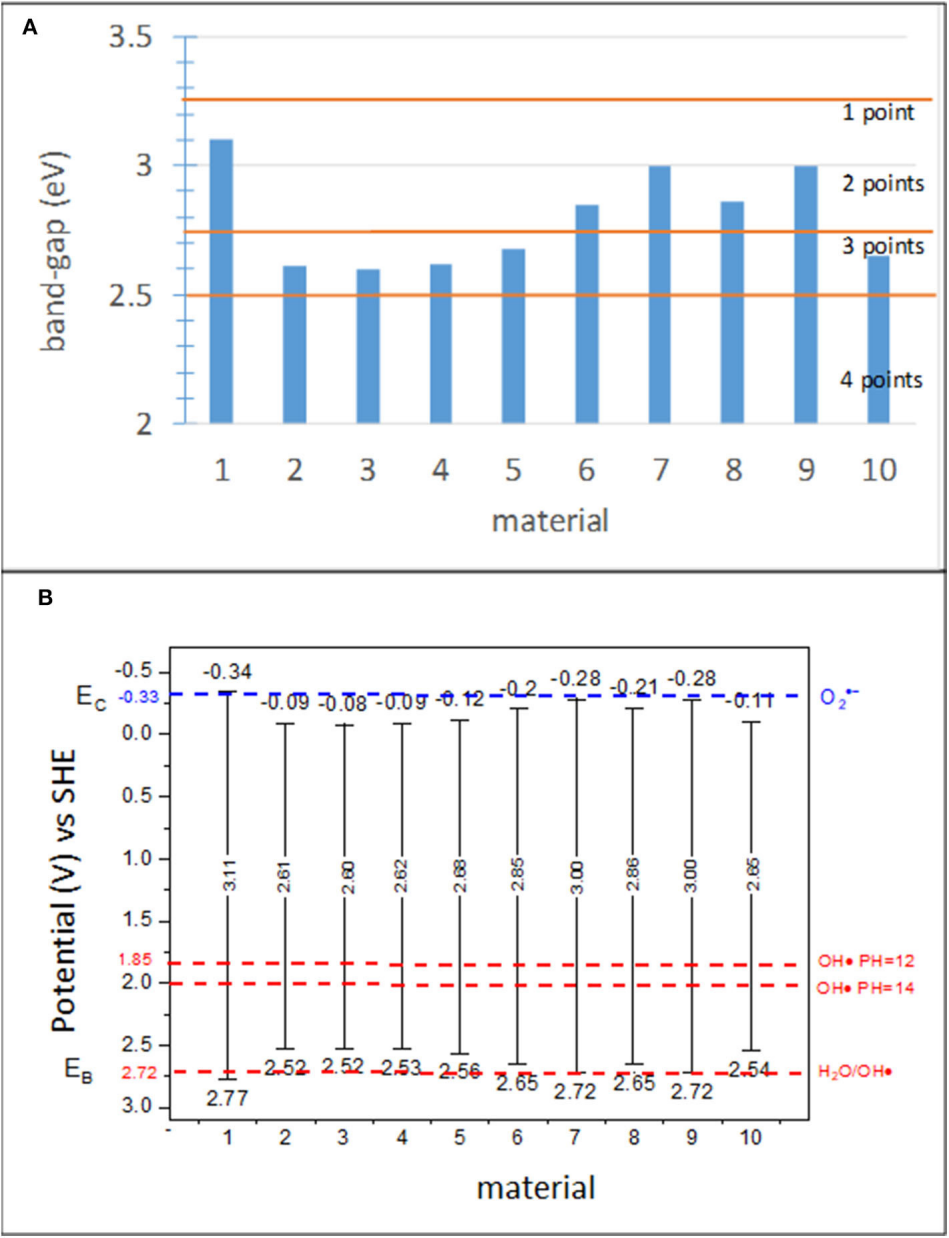
**(A)** *E*_*g*_ for various materials in the study and scoring ranges and **(B)** redox potentials of the valence band, *E*_*v*_, and conduction band, *E*_*c*_, determined with Equations 2 and 3 for the materials used in this study.

The band gap and position of the bands can be used to characterize the “potential effectiveness” of the photocatalyst. Although these two parameters were considered within the same subindicator, they were determined individually; thus, separate score ranges were ascribed to each of them. Both parameters have an important effect on the photocatalytic processes. The higher the band gap, *E*_*g*_, of a photocatalyst, the higher the photon energy that is required to activate a photocatalytic reaction. The *E*_*g*_ of TiO_2_ (anatase) is 3.2 eV. This energy determines the red bound of the spectral range, which can be absorbed by a photocatalyst (Gaponenko, 1998) at 388 nm. Unfortunately, according to Bird et al. (1983), the solar photon flux in this spectral range is <2.5% of the total flux. Actually, the situation is much more complex than this, as the amount of UV-A in the solar radiation reaching the Earth surface is extremely dependent on the latitude and time of the year (Folli et al., 2014). Significant efforts have been carried out to extend the absorption range and to increase the absorbed photon flux (Bengtsson et al., 2009; Rehman et al., 2009; Folli and Macphee, 2016). The scoring of this subindicator was designed to account for current and future photocatalysts, giving the photocatalysts with the red bound of the absorption spectrum in cyan (475–495 nm: 2.612–2.506 eV) a higher score, followed by the blue (450–475 nm: 2.757–2.612 eV) and violet (380–450 nm: 3.265–2.757 eV) ranges. The *E*_*g*_ values obtained for various materials studied in this work (Jimenez-Relinque et al., 2016) as well as the score ranges are shown in Figure 4A.

In addition to band gaps, the relative position of the conduction and valence band edges with respect to the redox potentials (at a given pH) for oxygen reduction and water oxidation, respectively, need to be considered. Band position dictates the thermodynamic feasibility of superoxide and hydroxyl radical generation, which are species highly involved in the oxidation of NOx, VOCs, and organics (Chatterjee and Dasgupta, 2005). The standard energy potentials of the involved half-reactions are as follows: −0.33 V for O_2_/${\text{O}}_{2}\cdot^{-}$ (Nosaka et al., 2011); +2.72 V for H_2_O/OH·, and +1.85–2.00 V for OH/OH· at an alkaline pH (12–14) (Folli et al., 2012). In addition, HO_2_·, H_2_O_2_, and OH· exhibit acid–base equilibrium involving protons; thus, they exist as ${\text{O}}_{2}\cdot^{-}$, ${\text{HO}}_{2}^{-}$, and O·^−^ at pH 4.8, 11.7, and 11.9, respectively (Nosaka et al., 2011).

The positions of the bands have to be determined experimentally using electrochemical methods (Gomes and Cardon, 1982; Sakai et al., 2004; Gelderman et al., 2007) and X-ray photoelectron spectroscopy (XPS) (Kraut et al., 1980; Kobayashi et al., 1994; Stefanov et al., 2008). In case it is not possible, Equations (2) and (3) (Ye et al., 2014) could be used to have an approximate value:

(2) $$\begin{array}{l} E_{c}\left(V\right)=\chi -{4.5-0.5\;E}_{g}, \end{array}$$

(3) $$\begin{array}{l} E_{v}\left(V\right)=E_{c}+E_{g}, \end{array}$$

where χ- is the absolute electronegativity of the semiconductor. The electronegativities of most materials can be found in the literature. Figure 4B shows a schematic of the bands with the values obtained for the 10 materials in this study using Equations (1) and (2) and χ = 5.72 eV for all samples, similar to the calculations of the theoretical values of the bands for anatase.

The authors recommend strongly the experimental determination of the band edges as Equations (2) and (3) are not accurate. In addition, the assumption that different TiO_2_ samples have the same absolute electronegativity is quite risky. On the other hand, the “optical” band gap (the one measured by UV-vis diffuse reflectance) is not necessarily the same as the “electronic” or “transport” band gap. The optical band gap is the threshold for photons to be absorbed, i.e., the excitation energy, which determines the onset of vertical inter-band transitions. The transport band gap is the energy necessary for creating an electron–hole pair (exciton) that is not bound together. As such, the optical band gap is smaller than the transport gap. To drive photocatalytic reactions, the real band gap is the transport one. In inorganic semiconductors there is little interaction between electrons and holes (i.e., very small exciton binding energy), and therefore, the optical and electronic band gaps are almost identical. Nevertheless, that is not always the case. In materials with high spatial localization of the valence and conduction states (e.g., organic semiconductors), it is possible for a photon to have just barely enough energy to create an exciton (bound electron–hole pair), but not enough energy to separate the electron and hole (which are electrically attracted to each other). In this situation, there is a significant difference between the optical and the transport band gap as the excitons have high binding energy.

The scores were defined according to the capability of the material to generate active species. The highest score was given to materials that can generate OH· during water oxidation and ${\text{O}}_{2}\cdot^{-}$ during the reduction of O_2_, whereas the lowest score corresponds to materials that can only generate OH· during the oxidation of OH^−^ ions.

#### Subindicator I1-2: Initial Photocatalytic Effectiveness for NO_x_ Abatement (ISO 22197-1:2007) R_NOx_ (%)

**Figure 5:**
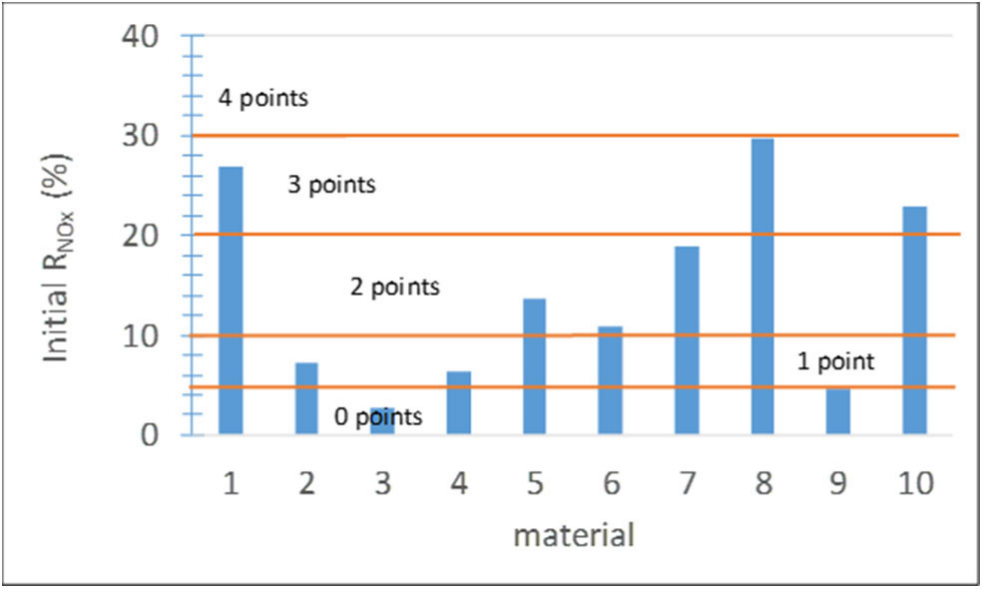
Initial NO_x_ removal effectiveness and corresponding score ranges.

This parameter can be determined according to ISO 22197-1:2007. Alternatively, a simplified method with shorter duration (usually 30 min) can be used as soon as the NO_x_ concentration at the outlet stabilizes, with the UVA light on. The results on site in the platforms and modeling with the environmental conditions are given in Jiménez-Relinque et al. (2019) and Hingorani et al. (2020). The score ranges (Figure 5) were established based on expert assessment considering the effectiveness of the commercial products on the market. The material photocatalytic activity is unacceptable (score 0) if the decrease in the NO_x_ concentration is equal to or smaller than 5%. Scores between 0 and 4 were assigned to the materials analyzed in the present study depending on their NO_x_ removal effectiveness (Figure 5).

#### Subindicator I1-3: Adhesion Strength to the Substrate

**Figure 6:**
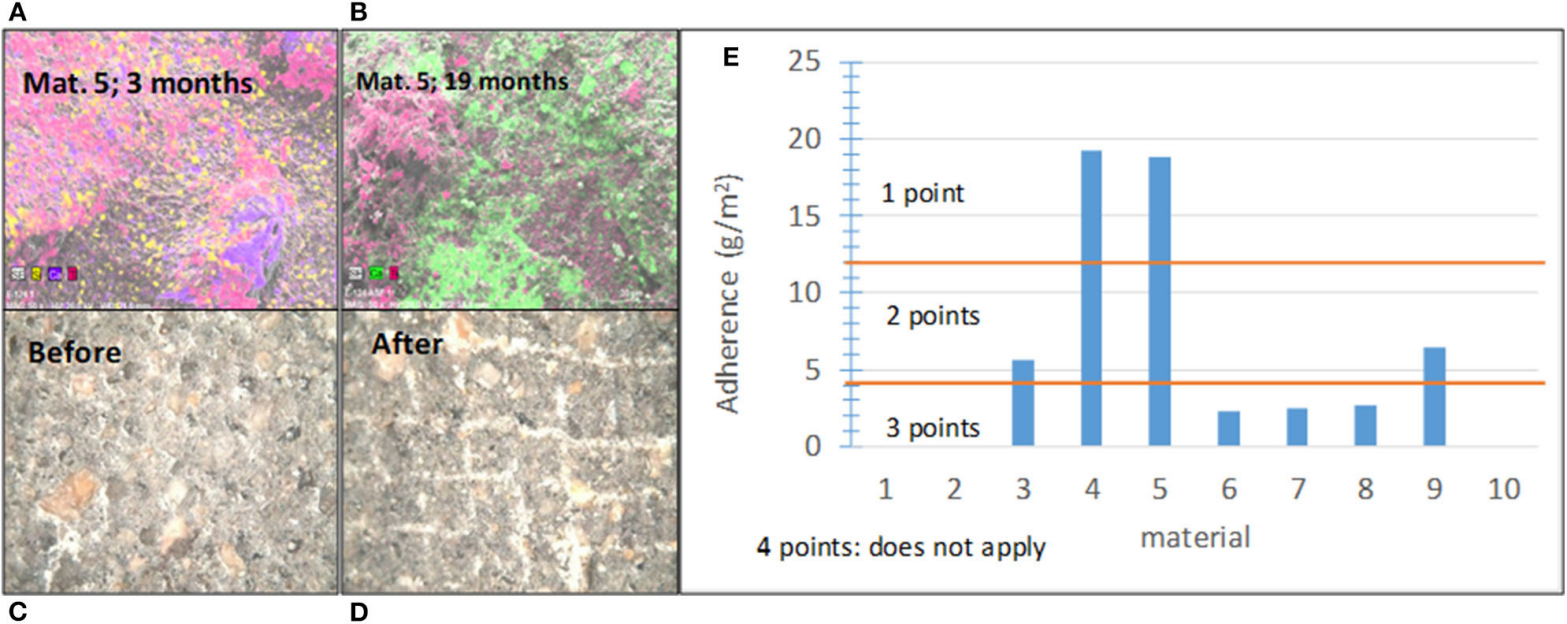
**(A)** SEM images and elemental maps of Ti (pink) for sample 5 after 3 months of outdoor exposure; **(B)** SEM images and elemental maps of Ti (pink) for sample 5 after 19 months of outdoor exposure; **(C)** images of the sample surface of material 4 before the adherence test; **(D)** images of sample surface of material 4 after the adherence test; and **(E)** summary of the results obtained for various materials in this study and the score ranges.

The adherence between the photocatalytic coating and substrate has a significant impact on the coating durability and overall performance (Sánchez et al., 2006; Chen and Poon, 2009a,b; Shan et al., 2010; Mendoza et al., 2015). In this study, it was proven that the aging-related decrease in the photocatalytic effectiveness is mainly associated with the wear and weathering of coatings with an insufficient adhesive strength to the underlying base pavement. This is illustrated in Figures 6A,B, which shows SEM images and elemental maps obtained using EDX for material 5 after (a) *t* = 3 months and (b) after *t* = 19 months of outdoor exposure. After 3 months, titanium (pink) can be observed on the whole sample surface (it is only missing in few zones). After 19 months, a much smaller amount of Ti, which was scarcely spread, was detected. Therefore, the adhesion strength was included in the conformity assessment as one of the subindicators.

Figures 6C,D shows the sample surface of material 4 before and after the cross-cut test. The score ranges were defined according to the results of this test related to behavior of the materials on the pilot-scale platforms. Figure 6E shows the established score ranges and results for the 10 materials. If the materials, which contain photocatalyst in the bulk, such as cementitious tiles with photocatalytic nanoparticles added to the mixture before casting, did not have an adhesion problem, they were assigned the highest score.

#### Subindicator I1-4: Carbonation

**Figure 7:**
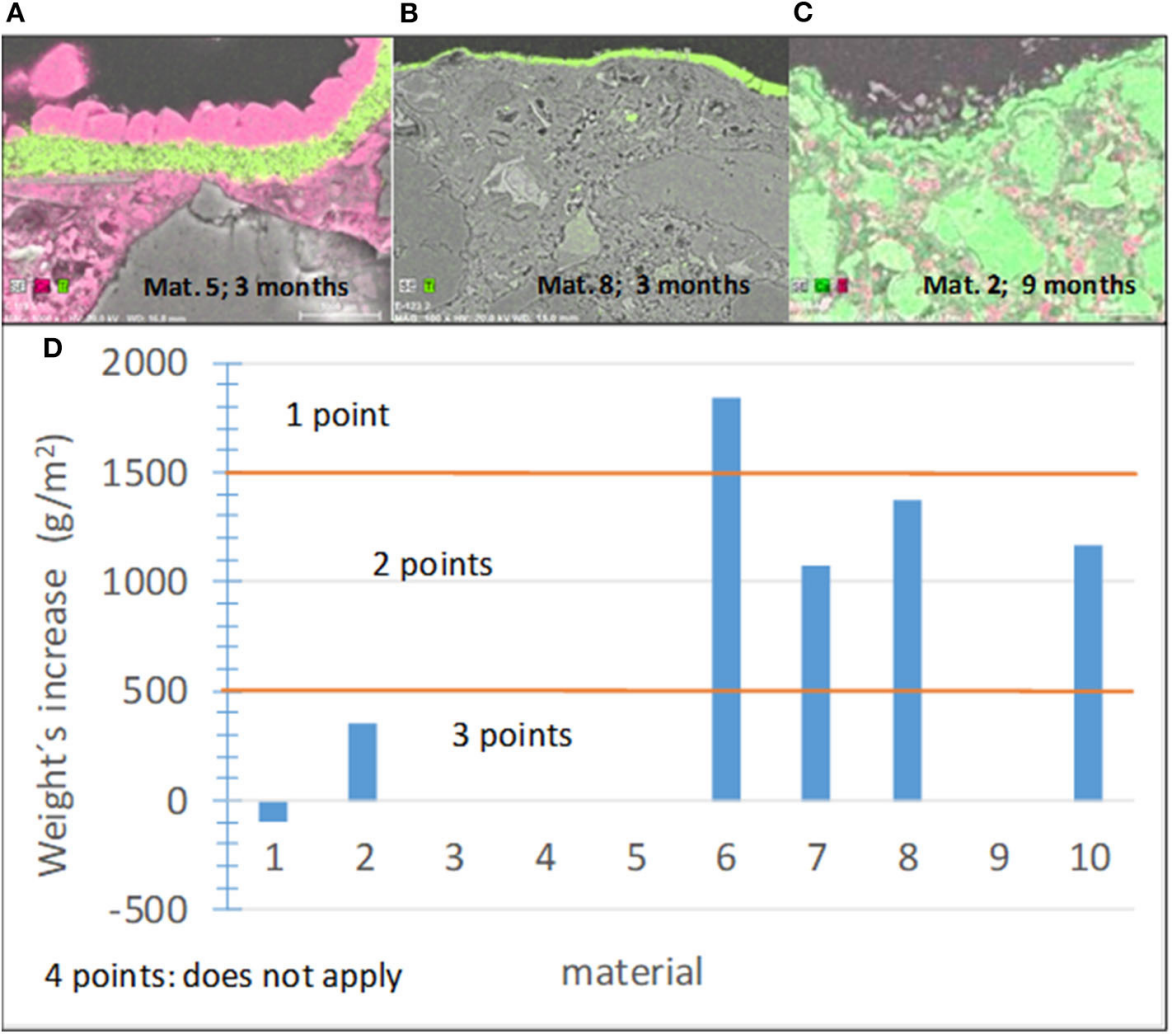
**(A–C)** Cross-sectional images of several materials showing different carbonation abilities. **(A,B)** Ti in red **(C)** Ti in pink. **(D)** Results of the carbonation tests and established score ranges.

Carbonation is a spontaneous reaction between Ca(OH)_2_, which forms during cement hydration, and atmospheric CO_2_, which results in calcite, CaCO_3_, formation on the sample surface. The opaque and dense calcite layer can block light, and the access of NO_x_ to active TiO_2_ centers reduces the photocatalytic effectiveness of the pavement with time. Several studies emphasized the effect of carbonation on the performance of photocatalytic materials (Karatasios et al., 2010; Diamanti et al., 2013; Fiore et al., 2013; Kaja et al., 2019; Lee and Kurtis, 2020). Given its dependence on Ca(OH)_2_, carbonation mainly occurs on cementitious pavements. As an example, Figures 7A–C shows the cross-sectional images of three materials used in this study and the elemental maps of Ti and Ca. The carbonation behavior of materials 5 and 8 differs, where the former shows a dense carbonate layer on the top of the photocatalytic coating, while carbonation cannot be detected in the latter. A non-compact layer can be observed in the case of material 2 (Figure 7C). Although the carbonate layer can have various structures, the criterion for the degree of the material susceptibility to carbonation was selected based on the mass gained during the accelerated carbonation test for the sake of simplicity, while precautions were taken to expose only the side of the sample with the photocatalytic coating to CO_2_. The specific mass increase per unit area, *M*_*C*_, after 1 week of the accelerated carbonation test was chosen as a quantitative parameter to assess this subindicator and establish the corresponding score ranges. These score ranges and the results obtained for the materials tested in this study are shown in Figure 7D. The highest score was assigned to materials that are not susceptible to carbonation.

### Main Indicator I2: Intrinsic Performance

The experimental results obtained in this study provide no evidence for any statistically significant variation in the mechanical properties or durability of pavement, which could be associated with the application of photocatalytic coatings or incorporation of photocatalytic materials into cementitious materials. This finding is in line with that of a previous study (Hernández-Rodríguez et al., 2019). The only pavement characteristic that is affected is the friction coefficient, which depends on many parameters such as the capacity to retain water, porous structure of the initial matrix, degree of hydrophilicity, and properties of the resulting photocatalytic surface.

#### Subindicator I2-1: Slip Resistance

**Figure 8:**
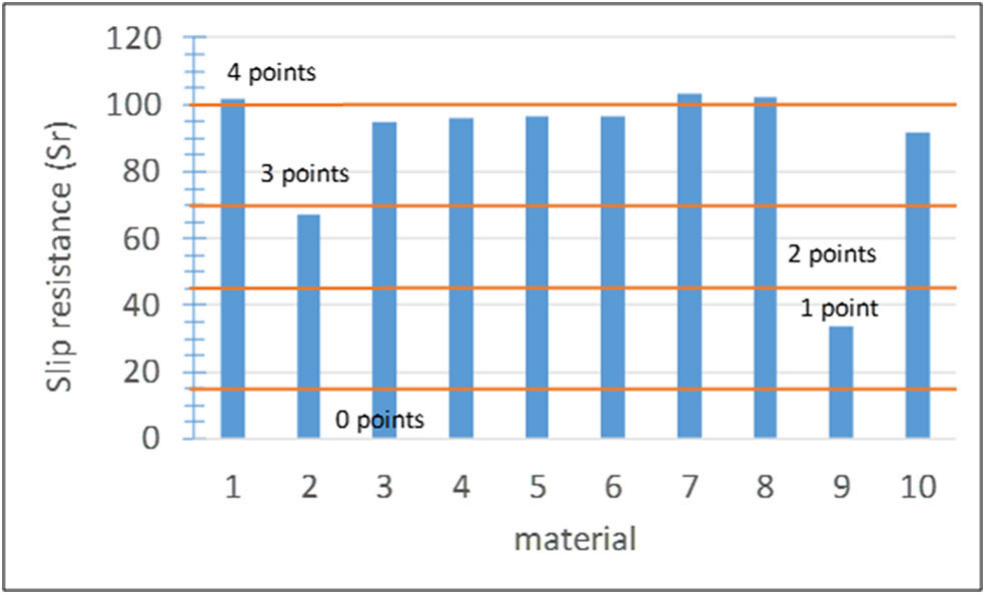
Results and score ranges for the slip resistance of the materials.

Previous studies showed that activated TiO_2_ increases the degree of hydrophilicity, which can lead to the formation of a thin water layer on the surface (effect underpinning self-cleaning of architectural glass; Fujishima and Zhang, 2006). This water film can affect the friction coefficient due to lubrication (Stiles, 1995) and therefore the slip resistance of the pavement. The slip resistance (*Sr*) was determined on both the laboratory and pilot scales using the pendulum slip meter according to standard EN 14231 adopted by the Spanish Technical Code of Buildings (CTE, 2006). Considering the classes specified in the standard, if *Sr* ≤ 15 (class 0 in the standard), the material is not safe and it is assigned score 0. Score 1 corresponds to classes 1 and 2 of the standard comprising dry and humid indoor pavements with a slope above 6%. Scores 2, 3, and 4 correspond to class 3 of the standard, which is the highest class suitable for humid outdoor pavements. The assessment of this indicator with only one subindicator differing from that of other indicators differed to avoid underestimation and discard materials that comply with the standard (see section Evaluation); values of 50, 70, 90, and 100% were directly assigned with scores 1–4, respectively. The score ranges adopted for this subindicator in this study are summarized in Figure 8.

### Main Indicator I3: Undesired Secondary Effects

Recently, the benefits of adding nanoparticles to construction materials have been questioned based on concerns regarding potential unwanted effects (Zhu et al., 2004; Chapman, 2006; Pacheco-Torgal and Jalali, 2011). In addition to advantages, several drawbacks of the use of nanotechnology have been reported, which are mainly related to the potential of nanoparticles to harm the environment after their release. The European Union has typified the exposure to nanoparticles as one of the emerging risks. Concerning TiO_2_, the Committee for Risk Assessment of the European Chemicals Agency (RAC) proposed to classify respirable titanium dioxide as carcinogen category 2 based on the inhalation of mixtures in powder form containing ≥1% titanium dioxide in the form of or incorporated in particles with aerodynamic diameters ≤10 μm, without giving specific concentration limits [Official Journal of the European Union (OJEU), 2020].

Although the toxicological studies of the effect of fine photocatalytic particles on humans are not yet conclusive (David Dankovic et al., 2011; Chen et al., 2016; Tobías et al., 2018), the results of many investigations provided experimental evidence of the capacities of fine and ultrafine solid particles, such as TiO_2_, to penetrate the air–blood barriers in mice (Yin et al., 2014) and deposit in organs (Kumar et al., 2014).

Another possible unwanted effect of the use of photocatalysts in construction is the potential formation of intermediates even more harmful than the original contaminant (Herrmann, 2005; Qourzal et al., 2008; Fotiou et al., 2013; Bloh et al., 2014).

In general, there is a gap of knowledge related to possible negative consequences of including nanoparticles in pavement materials. In the worst case, the potential release of such nanoparticles due to abrasion and erosion during its service life, disposal, and recycling might cause the secondary contamination of air, water, and soil, which might obscure the environmental benefits of reducing the NO_x_ and VOC concentrations.

#### Subindicator I3-1: Leaching of Ti

Under normal wear and weathering, TiO_2_ nanoparticles can be released to other media, such as water runoff, affecting aquatic ecosystems (Federici et al., 2007; Lovern et al., 2007), which are especially vulnerable due to the potential of mixing and dispersal. In this work, rain runoff was collected for each photocatalyst in workbench 2 on both platforms in plastic containers (Figure 3F) and the Ti and NO_3_ contents, pH, and conductivity were analyzed (Jiménez-Relinque et al., 2020). In addition to collecting rainwater, waterjet washing of the slabs simulating the procedure (adopted for street cleaning in Madrid) was carried out three times during outdoor exposure. Various Ti concentrations were detected in the water collected for all materials, with one exception. Therefore, Ti leaching was considered as one of the subindicators, which must be evaluated to assess the overall conformity of the photocatalytic pavement. Provided that the leaching behaviors of the materials under soft conditions (rain) differ from those under waterjet exposure, two different laboratory tests were carried out to simulate these conditions (section Laboratory Tests): leaching under soft conditions (I31-a) and waterjet leaching (I31-b).

**Figure 9:**
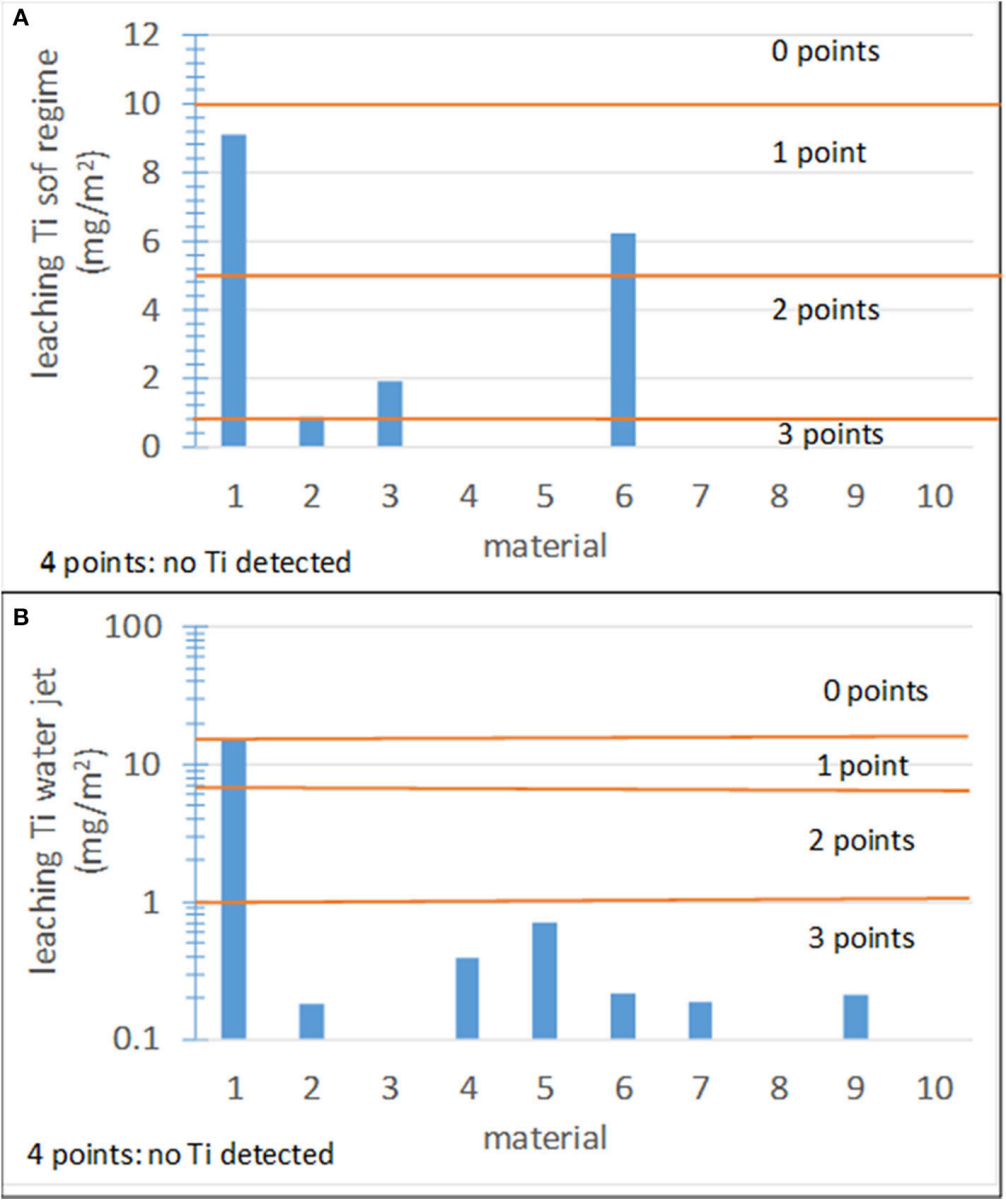
Leaching results for the 10 materials studied in this work and score ranges. **(A)** Leaching under soft conditions; **(B)** leaching under waterjet conditions.

Establishing the score ranges for Ti leaching is not easy because it should be based on toxicological studies, which remain inconclusive. Because thresholds for the admissible concentration of Ti in water cannot be found in the literature, the recommendations concerning concentrations of heavy metals summarized in the Drinking Water Parameter Cooperation Project supporting the revision of the Annex I Council Directive 98/83/EC on the Quality of Water Intended for Human Consumption (Drinking Water Directive) by the WHO were adopted as a guidance. The Ti concentration in the collected rainwater runoff is similar to the contents of arsenic and chromium (0.01 and 0.05 ppm, respectively) and lower than that of copper (2 ppm). Considering that these heavy metals are more toxic than Ti, the upper limit threshold was set to the highest Ti concentration (0.06 ppm) found in this study. Figures 9A,B shows the results for the 10 studied materials under soft and waterjet conditions, respectively. The corresponding score ranges are also shown.

#### Subindicator I3-2: Generation of Nanoparticles Aerosols

Non-exhaust sources of nanoparticle aerosols related to road transport were intensively studied on both road pavement simulators and in reality (Dahl et al., 2006; Thorpe and Harrison, 2008; Keuken et al., 2010; Kumar et al., 2013; Amato et al., 2014). Among these sources, tire–pavement contact and brakes are responsible for a significant proportion of the emitted nanoparticles with fine and ultrafine size ranges. A certain amount of nanoparticles can be emitted into the atmosphere by the weathering of nanofunctionalized photocatalytic building and construction materials (Hsu and Chein, 2007; Shandilya et al., 2015; Wohlleben and Neubauer, 2016; Koivisto et al., 2017; Nevshupa et al., 2020b). The results concerning nanoparticle emission of the photocatalytic material applied on a pilot scale in downtown Madrid as part of the LIFE-Photoscaling project are given in Nevshupa et al. (2020b). However, the particle generation mechanisms and influencing factors, including the road material, presence of embedded nanomaterials, road maintenance, and cleaning, are not sufficiently understood. Nevshupa et al. (2020a) made progress with respect to the more accurate quantification of the nanoparticle aerosol emission rate. They developed a new methodological framework based on the mass balance equation and analysis of the decay of the transient aerosol concentration. This methodology and the original experimental test rig Temis-1000, which was specially developed for this purpose, were used for the analysis of nanoparticle aerosol emissions from photocatalytic pavements in accelerated tests, which simulated tire–pavement abrasion. The tests were conducted both in the laboratory using cores extracted from photocatalytic pavements and on the platforms. A picture of Temis-1000 is shown in Figure 3D.

**Figure 10:**
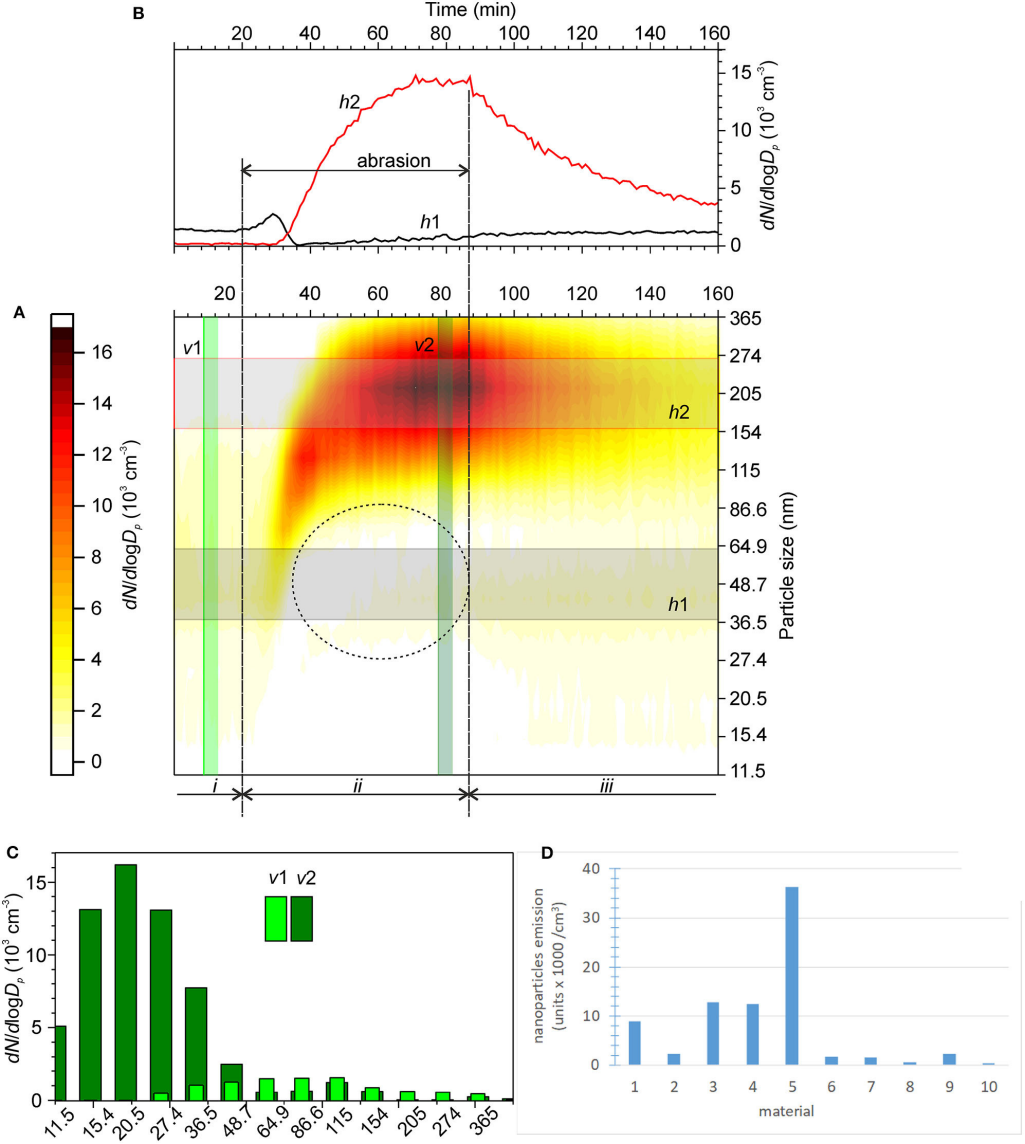
**(A)** Time series of the normalized concentrations of aerosol nanoparticles during the abrasion of a cement tile coated with photocatalytic material. *i*—stable residual aerosol concentrations in ambient air, *ii*—abrasion, and *iii*—transient particle concentration decay after the abrasion stopped; **(B)** averaged time series of the normalized concentrations of aerosol nanoparticles with the size ranges *h*1 and *h*2 denoted in **(A)**; **(C)** representative size distributions of aerosol nanoparticles in ambient air before the test (*v*1) and during abrasion (*v*2); and **(D)** results for the 10 materials studied in this work (units × 1,000/cm^3^).

As an example of the experimental results, Figure 10A shows the time series of the normalized concentrations of aerosol nanoparticles with different sizes obtained during the test. The residual aerosol concentration was measured before the test as benchmark (*i*). The average time series for various size ranges are shown in Figure 10B. Note that a decrease in the concentration of ultrafine particles can be frequently observed during the abrasion of cement tiles. This phenomenon might be associated with the scavenging of ultrafine residual particles by larger aerosol particles due to abrasion. However, this decrease did not affect the quantification of the emitted particles because different size ranges were used.

The number of emitted particles as a function of the experimental conditions was determined by integrating the time series considering the particle deposition velocities using the model developed by Nevshupa et al. (2020a):

(4) $$\begin{array}{l} N_{te,i}\left(t\right)=\int_{\varphi_{0}}^{t}K_{te,i}\left(t\right)\\ \;dt=V\left(C_{i}\left(t\right)-C_{i}\left(\varphi_{0}\right)+\frac{1}{\tau_{2,i}}\int_{\varphi_{0}}^{t}\left(C_{i}-C_{2,i}^{eq}\right)dt\right),\\ \;\varphi_{0}<t\leq \varphi_{1}, \end{array}$$

where *V* is the volume, *C*_*i*_ is the instantaneous concentration of nanoparticles within the size range *i*, φ_0_ is the time at which abrasion starts, φ_1_ is the time at which the abrasion ends, τ_2, *i*_ is the time constant of the particle deposition, and $C_{2,i}^{eq}$ is the stable aerosol concentration during abrasion.

The obtained value (units × 1,000/cm^3^) was used as the criterion for the assessment of the intensity of the nanoparticle aerosol generation by each pavement under the given experimental conditions. If the integration yields a negative value, for example, for ultrafine particles during the abrasion of cement tiles, the intensity is set to zero. The results obtained for the ten materials are shown in Figure 10D.

Two different cases were established for the same subindicator in this category considering the type of material and total absolute intensity of the particle emission in the size range <316 nm. Considering that the nanoparticle concentrations of most of the photocatalytic materials did not increase compared with those of the reference samples (78.5 and 1.18 unit × 1000 /cm^3^ for the reference asphalt and tile, respectively), rounded values based on these reference materials without a photocatalyst were used as criteria for admissible limits under the premise that they must not increase the nanoaerosols in the environment.

The scatter calculated for the values obtained with this method is ~20%, which is in line with the acceptable values for heterogeneous construction materials. The scores assigned to the ranges of *N*_*te*_ are listed in Table 2.

#### Subindicator I3-3: ${\text{NO}}_{3}^{-}$ Selectivity

**Figure 11:**
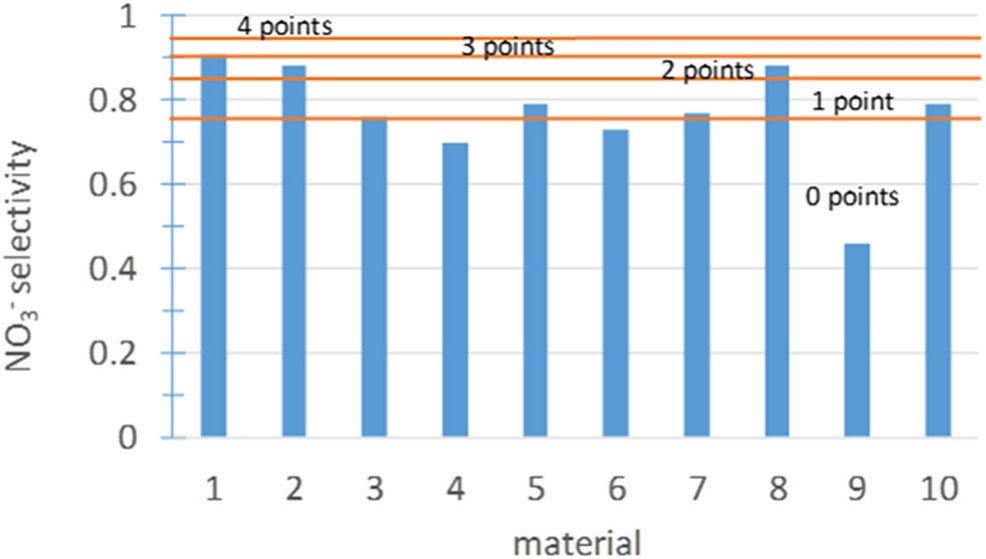
Nitrate selectivity and score ranges.

The selectivity of the photocatalyst during heterogeneous photocatalysis is another very relevant factor (Serpone and Emeline, 2005; Bloh et al., 2014; Kou et al., 2017). The target of the photocatalytic oxidation of NO and NO_2_ is the formation of nitrate or nitric acid. However, it is a complex process that involves several intermediate species. A complete diagram of the species involved is shown in Folli et al. (2018). In general, the photocatalytic oxidation of nitric oxide to nitrate proceeds in three individual one-electron transfer steps through the intermediate species nitrous acid (HNO_2_) and nitrogen dioxide (NO_2_; Bloh et al., 2014). Thus, it is necessary to assure that the final product is nitrate and that the photocatalytic process is not producing intermediate products, which are more harmful than NO. The nitrate selectivity was calculated as *S* = *R*_*NOx*_/*R*_*NO*_ according to Bloh et al. (2014). There is no need to perform new tests to determine this subindicator because the data were obtained from the tests of the NO_x_ abatement effectiveness. Based on expert assessment and considering the results obtained for the 10 materials in this study, the score ranges shown in Figure 11 were established.

## Assessment: Importance Factors

To classify the main and subindicators according to their importance, different weights were assigned according to expert criteria, as shown in Table 3. For example, an IF of 2.5 was assigned to the main indicator PPE. In the case of IP, provided that the materials that do not meet the established minimum based on the standard were discarded, the IF was established to be 1. A larger weight was assigned to the USE indicator because photocatalytic pavements are intended to improve the quality of the environment. Thus, if the material is susceptible to producing any adverse effect, it must be penalized to a greater extent. Hence, an IF of 5 was assigned, that is, two times higher than that of the PPE.

**Table 3:** Importance factors, I.F., for indicators and subindicators.

**Global indicators**	**I.F**	**Subindicators**	**I.F**
I1: PPE	2.5	I1-1-a	0.5
		I1-1-b	0.5
		I1-2	5.0
		I1-3	1.5
		I1-4	1.0
I2: IP	1	I2-1	1.0
I3: USE	5	I3-1-a	0.5
		I3-1-b	0.5
		I3-2	2.0
		I3-3	1.5

To validate the results of the present study, the IF and score ranges were presented in the final workshop of the project in June 2019 (version 0). In July 2019, the results were also made public on the internet (www.life-photoscaling.eu), opening a period of public surveys for 6 months. The comments and suggestions received from different stakeholders and experts were analyzed, and the IF and score ranges were amended accordingly. Tables 2, 3 present the updated values based on the expert assessment (version 1).

## Evaluation

**Figure 12:**
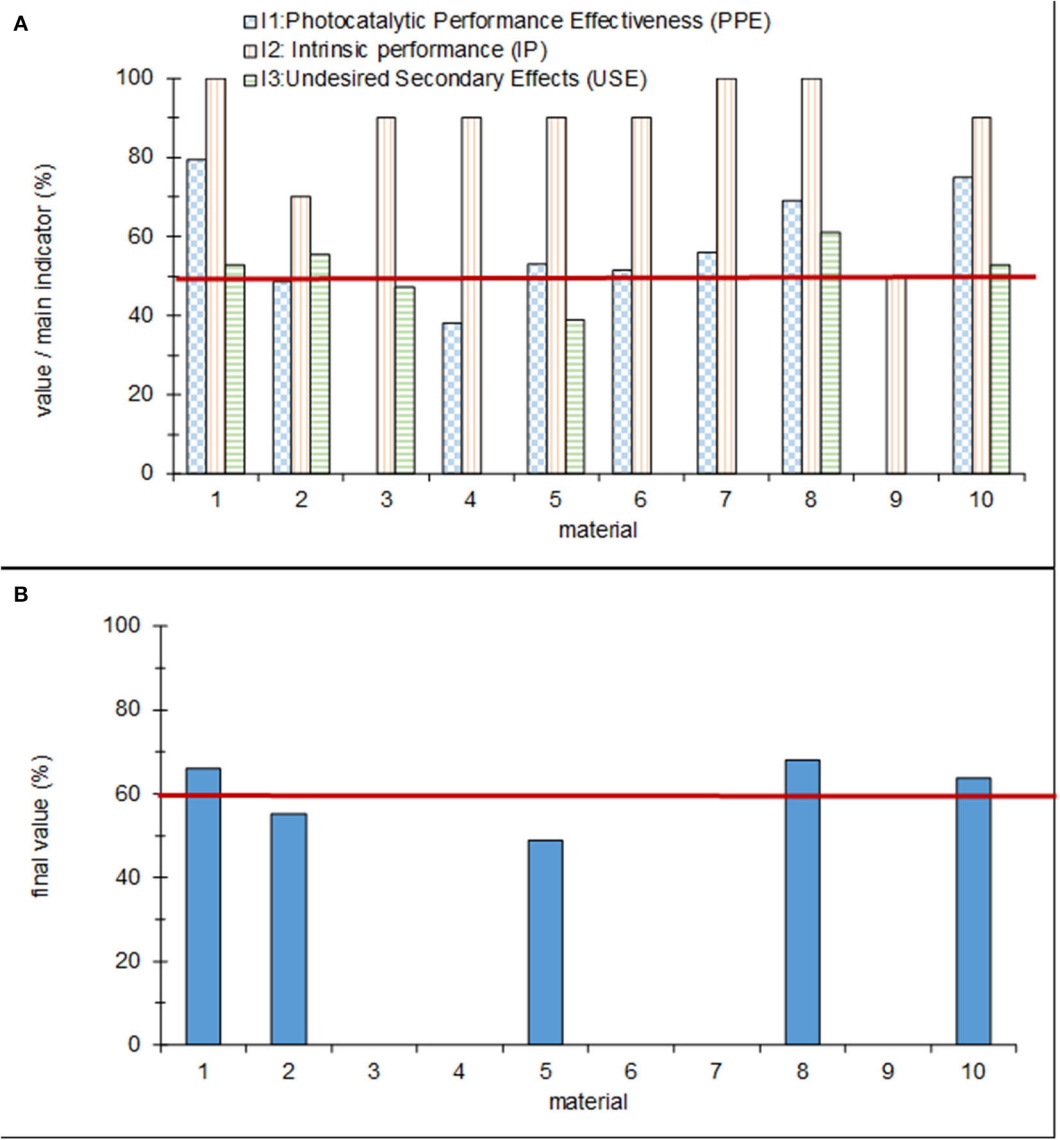
**(A)** Partial and **(B)** global values for each of the tested products. The red lines indicate the conformity assessment.

The results of the assessment procedure obtained for the 10 materials are shown in Figure 12. Figure 12A provides the scores obtained for the three main indicators, while the global result is shown in Figure 12B. The figures show that five of the materials (3, 4, 6, 7, and 9) were discarded for having a score of 0 for one of the subindicators. Based on the established framework, only three materials (1, 8, and 10) meet the established conformity criteria: a global assessment value of more than 60% and partial scores of the three main indicators above 50%. The results for the two remaining materials (2 and 5) are in the “gray” zone, which means that they must be improved before an application in the real world is possible.

The strengths and weaknesses of each material can be easily analyzed using the data shown in Figure 12. This analysis can help to determine the measures that are required to improve the materials' overall performance to comply with all requirements. Although significant efforts were made in the past to establish a common ground for the standardization of methods for testing photocatalytic pavements (Ifang et al., 2014), to the best of our knowledge, the holistic conceptual framework developed in this work presents a pioneer approach, which significantly improves the knowledge regarding the applicability and overall performance of photocatalytic pavement. This conceptual framework is a useful tool that can be adopted for the pre-standard assessment of photocatalytic materials.

## Conclusions

A new holistic conceptual framework for the indicator-based assessment of the performance and conformity of photocatalytic pavement materials was developed in this study. The process was developed using mechanisms on both the laboratory and pilot plant scales and by identifying three main indicators: (1) PPE, (2) IP, and (3) USE. Based on the characterization of 10 photocatalytic pavement materials, several key subindicators were established. To evaluate various material characteristics, traditional laboratory methods were used in combination with newly adapted or designed *ad-hoc* methods pursuing the objectives of a simple, time-efficient, and cheap characterization, which best represents real service conditions. The results of these tests were scored. The IFs were assigned to the main and subindicators based on expert criteria and were validated with a public survey. As a result, the “*PhotoScaling Decision Maker*” tool was developed, which can be used to evaluate each product both globally and partially according to three main indicators. This live tool was implemented in an open access online platform (www.life-photoscaling.eu) and enables knowledge-based decision-making regarding the selection of the most appropriate material under specific conditions and requirements.

## Data Availability Statement

All datasets generated for this study are included in the article/supplementary material.

## Author Contributions

MC conceived and directed the study and wrote the manuscript. EJ-R, RN, RH, and AC coordinated technically the parts of the work. FR programmed the tool available in the internet. MG carried out the tests. All authors contributed to the review, editing, and approval of the manuscript.

## Conflict of Interest

The authors declare that the research was conducted in the absence of any commercial or financial relationships that could be construed as a potential conflict of interest.
